# Effect of Natalizumab on sNfL and sGFAP Levels in Multiple Sclerosis Patients

**DOI:** 10.3390/ijms252313153

**Published:** 2024-12-07

**Authors:** Raquel Sainz-Amo, Alexander Rodero-Romero, Enric Monreal, Juan Luis Chico-García, Fernando Rodríguez-Jorge, Jose Ignacio Fernández-Velasco, Noelia Villarrubia, Jose Luis Veiga-González, Susana Sainz de la Maza, Jaime Masjuan, Lucienne Costa-Frossard, Luisa Maria Villar

**Affiliations:** 1 Neurology Department, Hospital Universitario Ramón y Cajal, Red Española de Esclerosis Multiple (REEM), Red de Enfermedades Inflamatorias (REI), ISCIII, Instituto Ramón y Cajal de Investigación Sanitaria, 28034 Madrid, Spain; raquelsainzamo@gmail.com (R.S.-A.); penry02@hotmail.com (E.M.); juanluis.chico.garcia@gmail.com (J.L.C.-G.); deterodriguez@hotmail.com (F.R.-J.); susanasmc85@hotmail.com (S.S.d.l.M.); jaime.masjuan@salud.madrid.org (J.M.); lufrossard@yahoo.es (L.C.-F.); 2 Immunology Department, Hospital Universitario Ramón y Cajal, Red Española de Esclerosis Multiple (REEM), Red de Enfermedades Inflamatorias (REI), ISCIII, Instituto Ramón y Cajal de Investigación Sanitaria, 28034 Madrid, Spain; alexander.rodero@salud.madrid.org (A.R.-R.); jfvelasco@salud.madrid.org (J.I.F.-V.); joseluis.veiga@salud.madrid.org (J.L.V.-G.)

**Keywords:** sNfL, sGFAP, SiMoA, multiple sclerosis, natalizumab

## Abstract

Natalizumab is a highly effective therapy for multiple sclerosis (MS). The aim of this study was to evaluate serum neurofilament light chain (sNfL) and serum glial fibrillary acidic protein (sGFAP) in patients with relapsing–remitting MS treated with Natalizumab. sNfL and sGFAP were analyzed at baseline, 6 and 12 months post treatment using the single-molecule array (SiMoA) technique. We recruited matched healthy controls for comparison. The study included 54 patients, with a median age of 33 years (Interquartile range (IQR), 29–41), with 32 women (60%) and 76 healthy controls. A decrease in sNfL was observed at 6 (67%, *p* = 0.005) and 12 (72%, *p* < 0.0001) months compared to baseline. After two years, six patients experienced evidence of disease activity (EDA-3). The remaining ones had no evidence of disease activity (NEDA-3). NEDA-3 presented a remarkable reduction in sNfL (*p* < 0.0001) and sGFAP (*p* = 0.01) after 6 months of treatment that continued to be observed after 12 months compared to baseline. EDA-3 only reached a significant decrease in sNfL after 12 months; there were no significant changes in sGFAP values. Natalizumab leads to a decrease in sNfL, which is higher and occurs earlier in NEDA-3 patients. Patients also showed a significant reduction in sGFAP levels, which was not observed in the EDA-3 group.

## 1. Introduction

Serum biomarkers play an important role in multiple sclerosis (MS), even more so since the development of new immune assays [1,2]. Neurofilaments are cytoskeletal proteins released into the cerebrospinal fluid and blood. Their quantification leads to the measurement of neuronal injury [3]. Serum neurofilament light chain (sNfL) has been validated in MS as a biomarker of disease activity. In addition, its elevation has been linked to disease progression [4,5,6,7].

Serum glial fibrillary acidic protein (sGFAP) is an intermediate filament present in the astrocytes [8]. Increases in sGFAP values have been related to future disability worsening, especially progression independent of relapse activity [9,10,11,12]. The application of these two biomarkers together seems to increase the ability to detect patients at risk of disease impairment [9,12,13].

Natalizumab is a monoclonal antibody used as a highly effective therapy for relapsing–remitting multiple sclerosis (RRMS). It is an IgG4 antibody that targets the α4 integrin, preventing harmful lymphocytes from entering the central nervous system. This is achieved by inhibiting the interaction between α4β1 integrin and vascular cell adhesion molecule-1 present in endothelial cells [14].

The way Natalizumab can make changes over time in sNfL and sGFAP values is not well known nowadays and evidence in the literature is scarce [15].

We aimed to evaluate both biomarkers in RRMS patients over their first year of treatment with Natalizumab. We compared their values with a cohort of matched healthy controls (HCs). In addition, we divided the patients into two groups according to the achievement of no evidence of disease activity status (NEDA-3) after two years of follow-up, to study differences in the evolution of sNfL and sGFAP values since the start of treatment.

## 2. Results

We incorporated 54 patients (32 women (60%)) into the study, all of whom initiated Natalizumab at Hospital Universitario Ramón y Cajal (Madrid, Sapin), a referral MS center. Clinical and demographic data of patients and HCs are shown in Table 1.

The median age (Interquartile range, IQR) of Natalizumab-treated patients was 33 (29–41) years. The annualized relapse rate (ARR) the year before treatment was 1 (1–2). The time from disease onset was 3.1 years (0.8–9.8). Twenty-six patients (48.1%) had not previously received disease-modifying treatment, and twenty-eight patients (51.9%) had received other treatment and needed a change due to lack of efficacy. Eight patients (14.8%) had at least 50 T2 lesions on baseline MRI; forty patients (74.1%) had gadolinium-enhancing lesions.

The data showed that 76% of patients had high (≥10 picograms/mL) sNfL values at treatment onset. Patients with high sNfL at treatment onset had more T2 lesions in MRI performed at baseline (*p* = 0.007). sNfL and sGFAP values were higher in RRMS patients at baseline compared to HCs (*p* ≤ 0.001 for both biomarkers).

**Figure 1:**
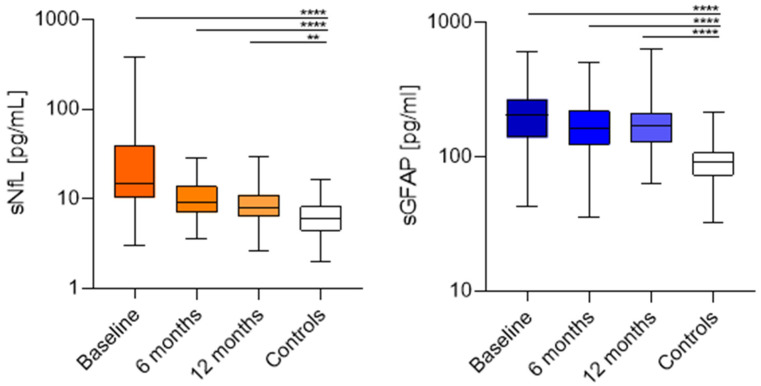
sNfL (picograms/mL) of RRMS patients and healthy controls at baseline, 6 and 12 months of Natalizumab; sGFAP (picograms/mL) of RRMS patients and healthy controls at baseline, 6 and 12 months of Natalizumab. ** (*p* = 0.006), **** (*p* < 0.0001).

A significant reduction in sNfL values was observed at 6 (67%, *p* = 0.005) and 12 (72%, *p* < 0.0001) months compared to baseline. However, the decrease between 6 and 12 months was not significant. Compared to HCs, differences persisted at 6 (*p* > 0.0001) and 12 (*p* = 0.006) months. We did not find significant changes in sGFAP values in the whole cohort (Figure 1).

Patients who switched from other DMTs had higher sNfL values (34.4 (14.2–73.4) vs. 12.5 (8.9–19.7), *p* = 0.0003) compared with naïve patients. These differences persisted at 6 months (12.9 (7–28.2) vs. 8.1 (6.7–10.9), *p* = 0.04), and disappeared at one year of follow-up (8.2 (6.4–14.3) vs. 8 (6.3–10), *p* = 0.4). However, we did not find differences in sGFAP values between these groups.

**Figure 2:**
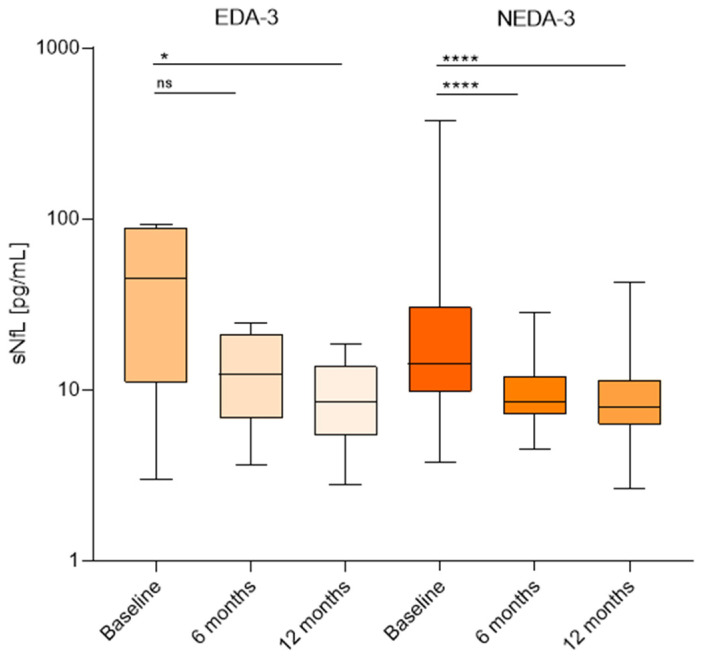
Comparison between sNfL (picograms/mL) values at baseline and after 6 and 12 months in EDA-3 and NEDA-3 patients after 2 years of Natalizumab. * (*p* = 0.01) **** (*p* = 0.0001) ns (non significant).

Patients were followed for two years. During this time, six patients experienced EDA-3. In four cases this was due to radiological activity, and in the other two to a clinical relapse. The remaining patients achieved NEDA-3. No significant differences were found in the baseline sNfL values of EDA-3 and NEDA-3 patients. These were 44.7 (11–89.2) picograms/mL and 12.4 (6.7–30.5) picograms/mL, respectively. NEDA-3 patients exhibited a reduction in sNfL after 6 months to 8.5 (7.2–12) picograms/mL, *p* < 0.0001, and after 12 months to 8 (6.2–11.4) picograms/mL, *p* < 0.0001, compared to baseline. By contrast, in EDA-3 patients the median sNfL value at six months of treatment was 15 (8.8–22.4) picograms/mL with no significant differences with baseline levels. Only after 12 months of treatment did we find a clear decrease in this group to 8.6 (5.4–13.7) picograms/mL, *p* = 0.01 (Figure 2).

**Figure 3:**
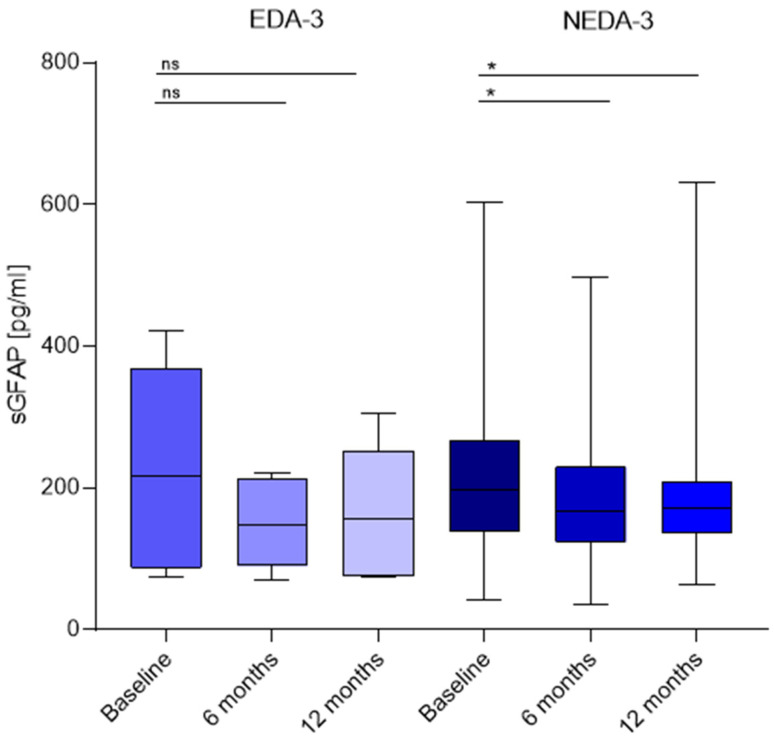
Comparison between sGFAP (picograms/mL) values at baseline and after 6 and 12 months in EDA-3 and NEDA-3 patients after 2 years of Natalizumab. * (*p* = 0.01, *p* = 0.02), ns (non-significant).

We did not find differences in baseline sGFAP between EDA-3 and NEDA-3 patients. The values were 216.9 (86.1–369) picograms/mL and 197.7 (138.7–266.9) picograms/mL, respectively. EDA-3 patients did not experience significant changes during the first year of treatment. Nevertheless, sGFAP values decreased in NEDA-3 patients after 6 months of treatment to 165.9 (123.7–229.5) picograms/mL, *p* = 0.01, and remained low after 12 months (171.9 (136.3–208.9) picograms/mL, *p* =0.02) compared to baseline (Figure 3).

## 3. Discussion

Acute axonal damage generated by highly inflammatory diseases can manifest as clinical (relapses) or radiological (increases in T2/gadolinium-enhancing lesions) activity. This is associated with high sNfL levels in RRMS patients [7]. According to this, sNFL levels are used as a biomarker for monitoring inflammation [7] and sGFAP seems to be linked to disease progression not associated with acute inflammation [9]. The combination of both biomarkers, sNfL, and sGFAP, may help to ameliorate the ability to detect patients at risk of worsening [10,13].

Early initiation of high-efficacy disease-modifying treatments in RRMS patients with high sNfL values at disease onset has been linked with a reduction in inflammatory activity and disease progression [7,13]. Nevertheless, there are only a small number of studies focused on variations in sNfL over time in RRMS patients who start a disease-modifying treatment. Furthermore, even fewer studies have analyzed both biomarkers (sNfL and sGFAP) together.

We aimed to analyze the role of sNfL and sGFAP in a group of highly active RRMS patients who started treatment with Natalizumab. First, we explored sNfL and sGFAP during the first year and compared the results to a cohort of matched HCs. We observed that sNfL decreased progressively, as described in other cohorts [16,17,18], but did not reach similar values to those of the HCs after a year of treatment. Natalizumab was administered every 4 weeks since, at that time, there was still no evidence of the 6-week dosing [19].

In another cohort [20], sNfL values were measured at 3 months and then after a year of treatment; consequently, they described nadir at one year of treatment. However, we observed that the decrease in sNfL values occurs mainly during the first six months, mostly in patients reaching NEDA-3 after follow-up. This decrease was maintained after one year of treatment. By contrast, the decrease was only observed after a year in EDA-3 patients, showing that an early reduction in sNfL levels was associated with an optimal response. This is in line with previous findings describing an association with sNfL ratio at 12 months and the risk of new MRI activity after two years of Natalizumab treatment [21].

Different data have been published about sNfL levels and their correlation with clinical and radiological activity in patients treated with Natalizumab. Thus, an increase in sNfL levels was linked to the recurrence of disease activity, defining these high levels as an early biomarker to predict the presence of disease before clinical or radiological signs appear [22]. From another perspective, a different research study [23] found no association between the wearing-off symptoms and sNfL and sGFAP levels in patients treated with Natalizumab, thus suggesting that it may not be associated with new disease activity, at least in all cases. However, research has focused more on the role of neurofilaments as predictors of the development of progressive multifocal leukoencephalopathy in patients treated with Natalizumab [24,25,26,27]. In our cohort, we observed that the reduction in sNfL values after six months of treatment could serve as a predictor of disease activity at two years, as only patients who reached NEDA-3 status after two years experienced a significant reduction in sNfL levels at this point.

Less evidence is available for sGFAP in Natalizumab-treated patients [15]. The possible effect of this drug on activated astroglia is not well known [14]. Our data showed no differences in this variable after 6 and 12 months of treatment in the entire cohort, but we found significant decreases at these points in the NEDA-3 group. These data strongly suggest that a reduction in sGFAP values in conjunction with that of sNfL levels can identify patients who will reach a NEDA-3 status in the long term during Natalizumab treatment.

The main limitation of our study was the sample size. These findings should be validated in larger, multicenter cohorts followed for a more prolonged period of time.

In conclusion, our data show that an early decrease in sNfL and sGFAP values could identify patients at low risk of disease activity during Natalizumab treatment.

## 4. Materials and Methods

### 4.1. Study Design

This was an observational study with prospective data collection, following the Strengthening the Reporting of Observational Studies in Epidemiology (STROBE) statement. Patients were recruited at the Hospital Universitario Ramón y Cajal in Madrid, Spain. We enrolled patients with RRMS who started Natalizumab treatment between March 2011 and August 2016. These patients were followed for two years. Treatment-naïve and previously treated patients were included. Patients received 300 mg of Natalizumab intravenously every 4 weeks. Age-, sex- and body mass index-matched HCs were recruited between August 2023 and February 2024.

### 4.2. Patient Consent

All patients and HCs signed an informed consent prior to participation. Anonymized data, which support the findings of this study, will be available to any qualified investigator upon request for 3 years following the publication of the study.

### 4.3. Data Collection

Clinical, radiological, and demographic variables were collected at onset. Experienced neurologists in the field conducted all Expanded Disability Status Scale (EDSS) evaluations every 3 months. Additional examinations were conducted in case of a relapse. A baseline MRI was performed within a month before treatment onset following established clinical protocols. Control MRI studies were performed annually.

### 4.4. Sample Collection

Patient blood specimens were collected just before initiating Natalizumab treatment and again at 6 and 12 months after that. Serum sample aliquots were stored at −80° until they were processed.

### 4.5. Serum sNfL and sGFAP Quantification

sNfL and sGFAP were quantified using an HD-X instrument (Quanterix, Lexington, MA, USA) with the single-molecule array (SIMoA) technique (Quanterix, Billerica, MA, USA). We employed a Neurology 2-Plex B Kit (Quanterix, Billerica, MA, USA), following the manufacturer’s instructions. The mean inter- and intra-assay coefficients equaled 5.6% and 4.4% for sNfL and 5.8% and 5% for sGFAP, respectively. The research team handling the evaluation of the serum samples remained unaware of the clinical data.

### 4.6. Definitions

We used the 2017 McDonald criteria for diagnosis [28]. Disability was assessed with the EDSS score [29]. Confirmed disability worsening was defined as a rise of at least 1.5 points in the EDSS if the baseline score was 0, an increase of at least 1 point if the previous EDSS was between 1 and 5, and a minimum 0.5 point increase for patients with a baseline EDSS of 5.5 or higher [30]. NEDA-3 was defined as the absence of relapses, disability worsening and new and/or enlarged T2 lesions or gadolinium-enhancing lesions on MRI. Patients experiencing a relapse, MRI activity, or an exacerbation of neurological disability were classified as having evidence of disease activity-3 (EDA-3) [31].

The cut-off applied for sNfL and sGFAP levels was established at the 90th percentile value of the corresponding HC, which was 10 picograms/mL for sNfL and 140 picograms/mL for sGFAP, in line with the benchmarks used in previous studies [5,7,31,32,33].

### 4.7. Statistical Analyses

Descriptive analyses were summarized using absolute and relative proportions for categorical variables, and differences were examined using χ² or Fisher’s exact test. The median with an Interquartile range (IQR) was employed to describe continuous variables, and associations between groups were evaluated using the Friedman and Mann–Whitney U tests. We performed statistical analyses using the GraphPad Prism 9.0 software (GraphPad Prism Inc., San Diego, CA, USA). All tests were two-tailed, and a significance level of *p* < 0.05 was deemed significant.

## Figures and Tables

**Table 1:** Baseline data.

	RRMS (n = 54)	HCs (n = 76)	*p* Value
Age (years)	33 (29–41)	31 (26–46)	n.s.
Female/Male	32/22	47/29	n.s.
Body mass index	25.3 (21.6–28.1)	22.5 (20.5–24.7)	n.s.
Time from disease onset (years)	3.1 (0.8–9.8)		
EDSS score	2 (1.5–2.5)		
ARR 1 year before	1 (1–2)		
Previous treatment			
None	26 (48.1%)		
Platform	27 (50%)		
Orals	1 (1.8%)		
Monoclonal antibody	0		
T2 lesions (<10, 10–50, >50)	7(13%), 39(72.2%), 8 (14.8%)		
Number of gadolinium-enhancing lesions	1 (0–4)		
Patients with gadolinium-enhancing lesions	40 (74.1%)		
Oligoclonal IgG bands	52/53 (98.1%)		
Oligoclonal IgM bands against lipids	43/53 (81.1%)		
sNfL (picograms/mL)	15 (10.2–39)	6.11 (2–8.5)	*p* < 0.0001
sGFAP (picograms/mL)	203.5(138.1–268.4)	91 (72.6–109)	*p* < 0.0001

Abbreviations: n, number of patients and controls; RRMS, relapsing–remitting multiple sclerosis; HCs, healthy controls; EDSS, Expanded Disability Status Scale; ARR, annualized relapse rate, n.s. non-significant. Platform treatments: interferon b and glatiramer acetate; oral drugs: dimethylfumarate, fingolimod, teriflunomide. Continuous variables are shown as median (IQR) and categorical variables as numbers (%).

## Data Availability

The raw data can be obtained from the corresponding author upon reasonable request in the three years following the manuscript’s publication.
